# Quantitative Assessment of New Frontiers in Dermatochalasis and Periorbital Hyperpigmentation Treatment: The Role of Cross-Linked Porcine Collagen

**DOI:** 10.1007/s00266-024-04407-1

**Published:** 2024-10-15

**Authors:** Andy Deng-Chi Chuang, Erh-Ti Lin, Bing-Qi Wu, Meng-En Lu, Hsiu-Mei Chiang, Pai-Nien Chu, Bor-Shyh Lin, Chang-Cheng Chang

**Affiliations:** 1 Department of Surgery, Chang Gung Memorial Hospital, Taoyuan, Taiwan; 2 Department of Orthopedics, China Medical University Hospital, No. 2, Yude Rd., North Dist., Taichung, 404327 Taiwan; 3 Department of Education, China Medical University Hospital, No. 2, Yude Rd., North Dist., Taichung, 404327 Taiwan; 4 Department of Cosmeceutics and Graduate Institute of Cosmeceutics, China Medical University, Taichung, Taiwan; 5 Younger Cosmetic Hospital, Beijing, China; 6 Institute of Imaging and Biomedical Photonics, National Chiao Tung University, Tainan, Taiwan; 7 Aesthetic Medical Center, China Medical University Hospital, No. 2, Yude Rd., North Dist., 404327 Taichung, Taiwan; 8 Division of Plastic and Reconstructive Surgery, Department of Surgery, China Medical University Hospital, No. 2, Yude Rd., North Dist., Taichung, 404327 Taiwan; 9 Institute of Imaging and Biomedical Photonics, National Chiao-Tung University, Hsinchu, Taiwan

**Keywords:** Dermatochalasis, Periorbital hyperpigmentation, Collagen dermal filler, Skin analysis system

## Abstract

**Background:**

Collagen dermal fillers have shown efficacy in addressing age-related changes in facial appearance. However, their potential in rejuvenating the periorbital region remains unexplored. The aim of this study was to evaluate the effectiveness, clinical safety, and patient satisfaction associated with the utilization of collagen dermal fillers in individuals with dermatochalasis and periorbital hyperpigmentation.

**Methods:**

This study was reviewed and approved by the institutional review board of China Medical University Hospital (IRB No. CMUH107-REC2-157). Adults diagnosed with dermatochalasis or periorbital hyperpigmentation received periorbital injections of a sterile cross-linked highly-purified specific antigen free porcine dermal collagen (FACIALGAIN® Collagen Implant with Lidocaine, Sunmax Biotechnology Co. Ltd., Taipei, Taiwan) and were assessed for hydration, elasticity, pigmentation index, redness index, lightness value, and density with the DermaLab® Combo Multiparameter Skin Analysis System (Cortex Technology, Hadsund, Denmark) and Cutometer® Dual MPA 580 (Courage+Khazaka electronic GmbH, Köln, Germany). Data was collected prior to injection and at 1 week, 4 weeks, and 12 weeks after injection. Patient satisfaction on volume augmentation, decrease in hyperpigmentation, persistence, and overall satisfaction were also recorded.

**Results:**

A total of 12 samples each were recruited for dermatochalasis and periorbital hyperpigmentation. For dermatochalasis subjects, hydration of the periorbital tissue significantly increased at week 1 and 4 (*p* = 0.011 and *p* = 0.015). Elasticity decreased by week 4 and persisted until week 12 (*p* = 0.001 and 0.014). For periorbital hyperpigmentation patients, lightness value increased significantly starting week 1 (*p* = 0.016), tapering off at week 12. Elasticity decreased by week 4 and persisted until week 12 (*p* = 0.002 and *p* = 0.002). Median overall patient satisfaction was 4 out of 5 for the dermatochalasis group and 4.5 out of 5 for the periorbital hyperpigmentation group, with a mild but insignificant decrease by week 12.

**Conclusions:**

DermaLab® Combo and Cutometer® Dual MPA 580 are considered effective methods for evaluating patients undergoing dermal filler injections. The utilization of cross-linked porcine collagen dermal filler injections can serve as a minimally invasive approach to enhance skin laxity in dermatochalasis and address discoloration in periorbital hyperpigmentation. However, it is important to note that regular treatments may be necessary to sustain the desired outcomes.

**Level of Evidence IV:**

This journal requires that authors assign a level of evidence to each article. For a full description of these Evidence-Based Medicine ratings, please refer to the Table of Contents or the online Instructions to Authors www.springer.com/0026.

## Introduction

A variety of endogenous and exogenous factors, including age, ultraviolet light exposure, fatigue, and genetics, contribute to alterations in the periorbital tissue’s appearance [1]. Dermatochalasis, periorbital fat herniation, deep tear troughs, or periorbital hyperpigmentation may subsequently manifest. In individuals with lighter skin tones, these alterations are prominent and can be easily noticed, leading to psychosocial implications [2]. The past decade has witnessed a growing trend in the utilization of dermal fillers for periorbital rejuvenation, driven by the quest for a treatment option that is both effective and minimally invasive [3, 4].

In the context of facial and periocular skin rejuvenation, collagen and hyaluronic acid are the most frequently utilized dermal fillers [5, 6]. Traditionally, hyaluronic acid has been the predominant choice in the dermal filler market because of its comparatively low immunogenicity and superior durability in comparison to collagen [6]. In the case of hyaluronic acid, post-procedural malar edema, limited response to hyperpigmentation, or the Tyndall effect may be observed [7–10]. In recent years, there has been a resurgence in the popularity of collagen-based dermal fillers, attributed to the introduction of advanced collagen preparation techniques like cross-linking and a transition from bovine to porcine dermal collagen. The effectiveness of porcine collagen fillers in addressing prominent nasolabial folds and facial actinic scarring has been demonstrated in the facial region [11–14]. Limited research has been conducted on the efficacy of dermal fillers, including collagen and hyaluronic acid, for treating dermatochalasis or periorbital hyperpigmentation. The presence of thinner overlying skin and less dense periorbital tissue can result in more noticeable post-procedural edema, Tyndall effect, or potentially worsened hyperpigmentation following the use of hyaluronic fillers [10]. Therefore, collagen injection may be the preferred option for treating dermatochalasis and periorbital hyperpigmentation because of its relatively lower incidence of adverse effects. In our research, we aim to establish the effectiveness of collagen injection in addressing dermatochalasis and age-related changes in the eye. We intend to support our findings with quantitative data suitable for statistical analysis, obtained through evaluations using skin analysis tools like DermaLab Combo® by Cortex Technology, Soft Plus® by Callegari, and different probe systems by Courage + Khazaka Electronic GmbH.

## Materials and Methods

### Patient Selection

This study underwent review and approval by the institutional review board of China Medical University Hospital (IRB No. CMUH107-REC2-157). Subjects were enrolled at a solitary medical center from August 2018 to July 2019. Consenting patients aged 18 and above underwent evaluation by board-certified plastic surgeons, who diagnosed dermatochalasis or periorbital hyperpigmentation as appropriate. Patients exhibiting severe dermatochalasis, who would derive greater benefit from surgical intervention, were excluded. Patients with a history of allergic reactions to collagen injections or other dermal procedures, as well as those who had undergone recent surgery or were currently undergoing treatment for dermatochalasis or periorbital hyperpigmentation within the preceding 6 months, were excluded from the study.

### Materials

Glutaraldehyde cross-linked porcine dermal type I atelocollagen infused with lidocaine (FACIALGAIN® Collagen Implant with Lidocaine) was sourced from Sunmax Biotechnology Co. Ltd. (Taipei, Taiwan). DermaLab® Combo, a multi-parameter skin analysis system, was procured from Cortex Technology (Hadsund, Denmark), and the skin elasticity probe (Cutometer® Dual MPA 580) was obtained from Courage + Khazaka electronic GmbH (Köln, Germany).

### Methods

Following the recruitment process, high-resolution photographs of the face of each patient were captured, and baseline measurements were documented using skin analytical systems. Skin hydration was evaluated through the measurement of skin conductance using a specific probe. An elevation in electrical conductance signifies higher water content in the skin. Skin density was evaluated using an ultrasound probe to measure the intensity of the ultrasound signal, and a density score was automatically calculated based on this intensity. Skin color and lightness were assessed using a color sensor and quantified in CIEL*a*b* color values. The viscoelasticity of the skin was assessed using the Cutometer® MPA 580. The designated skin area was drawn into the airtight chamber of the elasticity probe through the application of negative air pressure. The distance that the skin regained within one second following the release of negative pressure was subsequently quantified and presented as a percentage of the overall skin deformation.

Measurements were conducted at baseline, as well as in weeks 1, 4, and 12 during the follow-up period. Measurements were recorded individually for each eye. The study involved measuring the average skin density in the regions with the thickest dermis in the lower eyelid and tear trough of each eye. Hydration and viscoelasticity were assessed in the tear trough region. The CIELAB color space was directly measured below the iris at the level of the infraorbital foramen. Probe positions were recorded, and subsequent measurements were conducted at the identical site.

Following the procedure, patients are required to complete a questionnaire assessing their satisfaction regarding the perceived volume augmentation, reduction in pigmentation, longevity of the dermal filler, and overall contentment. The satisfaction questionnaire necessitates that patients select a score on a scale of one to five, where one indicates extreme dissatisfaction and five indicates extreme satisfaction. The participants were requested to attend follow-up appointments at 1, 4, and 12 weeks post the initial injection. Measurements using DermaLab® Combo and Cutometer® Dual MPA 580 are repeated during subsequent follow-up appointments, in conjunction with patient satisfaction surveys.

Statistical analyses were conducted using SPSS (version 25; IBM SPSS Inc., Chicago, Illinois). Measurements obtained during weeks 1, 4, and 12 following the procedure were compared to the baseline measurements. The Shapiro-Wilk test was employed to assess the normality of the data. Normally distributed data were assessed using the paired Student t-test, whereas non-normally distributed data were examined using the Wilcoxon signed rank test. The Kruskal-Wallis test was utilized to identify any statistically significant differences in the responses gathered from patient satisfaction questionnaires. A significance level of 0.05 was employed to ascertain the statistical significance of the findings.

Prior to enrollment, all patients provided informed consent for their participation in this study. This study underwent review and approval by the institutional review board of China Medical University Hospital (IRB No. CMUH107-REC2-157), and it was conducted in accordance with the principles set forth in the Declaration of Helsinki.

### Techniques

**Fig. 1:**
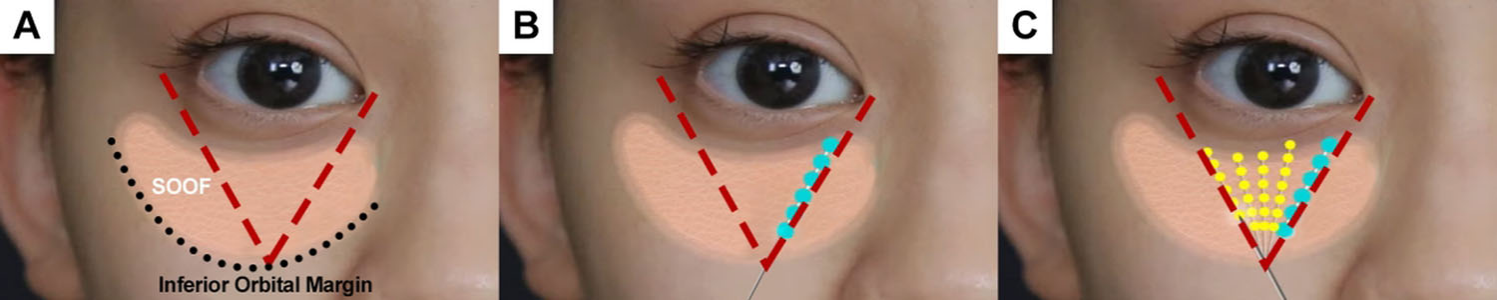
**A** Boundaries of injection (red dashed lines) drawn from the inferior orbital margin (black dotted line) to the medial and lateral canthi. The suborbicularis oculi fat (SOOF) layer deep to the skin is shown. **B** Injections are made into the SOOF layer within the marked boundaries in a retrograde manner. **C** Between four to five injections are done within the marked boundaries, depending on the response

Prior to injection, the sites are sterilized with alcohol swabs. An imaginary line extends from the medial canthus to the inferior margin of the orbital rim, while a second line runs from the lateral canthus to the inferior margin, creating a 90-degree angle between the two lines (Fig. 1A). At the point of intersection, an injection is administered using a 27-gauge needle that is 38 millimeters in length. The injection angle ranges from 15 to 25 degrees relative to the skin, targeting the suborbicularis oculi fat (SOOF) layer (Fig. 1B). The needle is oriented superiorly, and injections are administered in a fanning pattern within the confines delineated by the medial and lateral canthi (Fig. 1C). Injections are administered in four to five fractions in a retrograde fashion. An additional subcutaneous injection in four to five fractions with the needle parallel to the skin may be administered if the texture of the overlying skin is more susceptible to wrinkling and irregularity. A volume of dermal filler ranging from 0.5 to 1.2 milliliters was injected into the treated areas around each eye, with the specific volume determined through real-time inspection. After the injection, the treated areas are gently massaged until no palpable irregularities can be palpated.

## Results

**Fig. 2:**
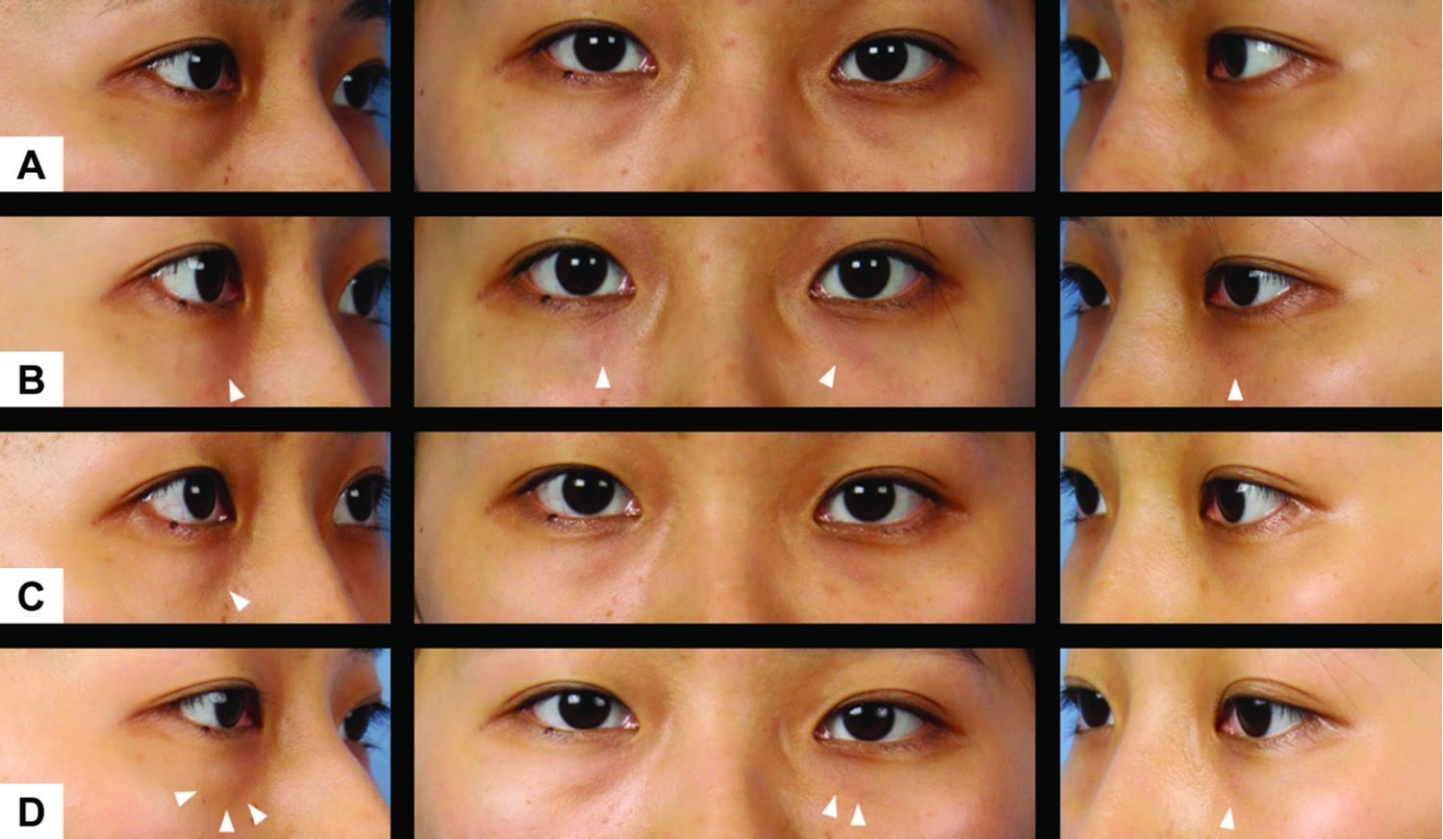
An example of a subject with periorbital hyperpigmentation **A** before receiving treatment, **B** 1 week after injection, **C** 4 weeks after injection, and **D** 12 weeks after injection. A noticeable increase in brightness of skin tone is observed over the infraorbital area surrounding the injection sites (white arrows). The effects seemingly persist throughout the 12 weeks

**Fig. 3:**
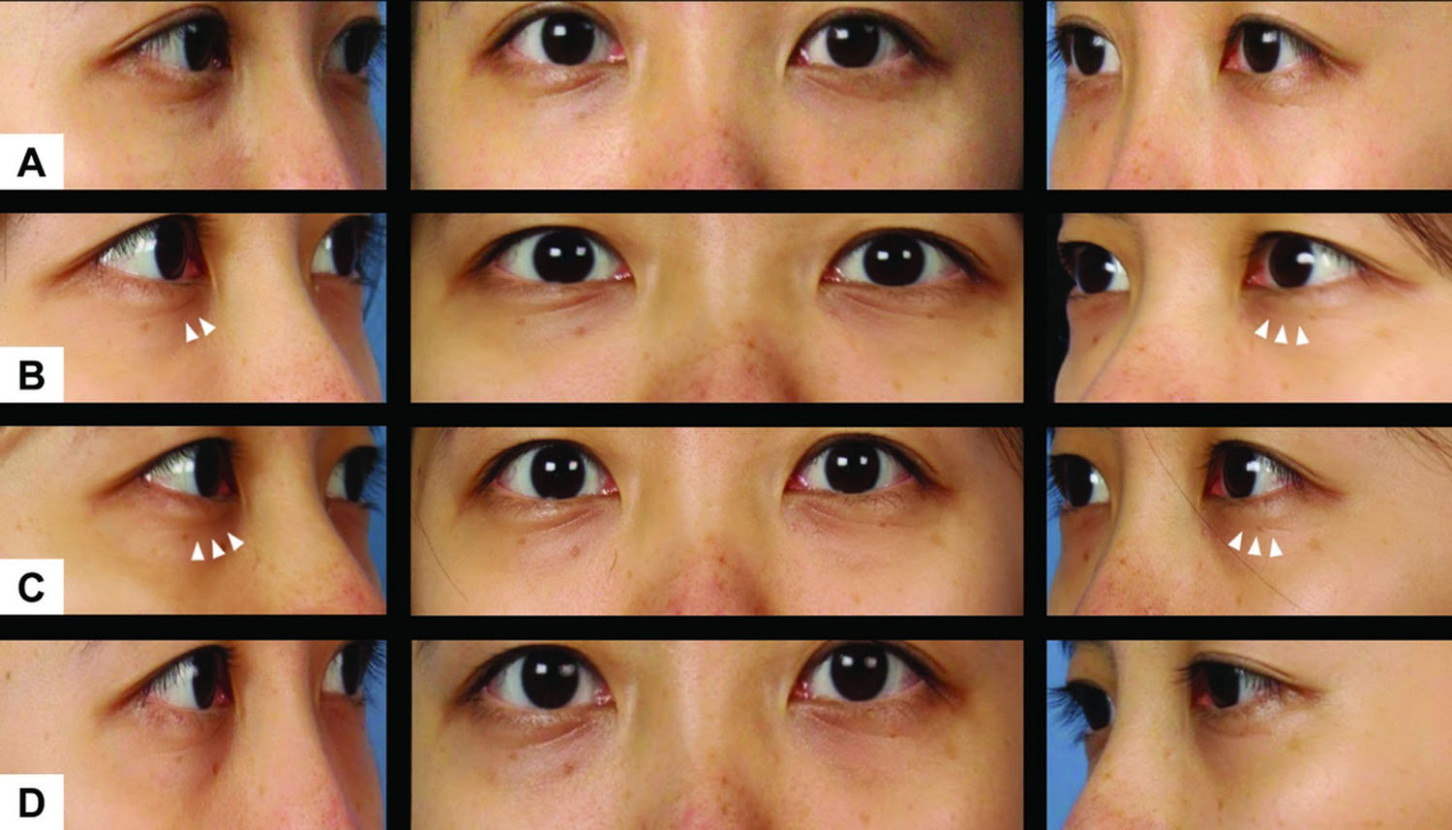
An example of a subject with dermatochalasis **A** before receiving treatment, **B** 1 week after injection, **C** 4 weeks after injection, and **D** 12 weeks after injection. The decreased laxity in the periorbital tissue at the injection sites are perceivable initially (white arrows) but becomes less obvious at week 12

A cohort of 20 patients was enrolled, comprising 10 individuals diagnosed with periorbital hyperpigmentation and 10 individuals diagnosed with dermatochalasis. Four patients from each group were lost to follow-up among the 20 patients included in our study. Among the remaining six patients in each group, five were female, and one was male in both groups. The average age of the participants in the periorbital hyperpigmentation group was 28.3 years (median: 26.5 years), whereas the average age in the dermatochalasis group was 33.8 years (median: 30 years). In the periorbital hyperpigmentation group, all six patients underwent supplementary subcutaneous injections in both eyes, whereas only two out of six patients in the dermatochalasis group received additional subcutaneous injections (Figs. 2 and 3).

**Fig. 4:**
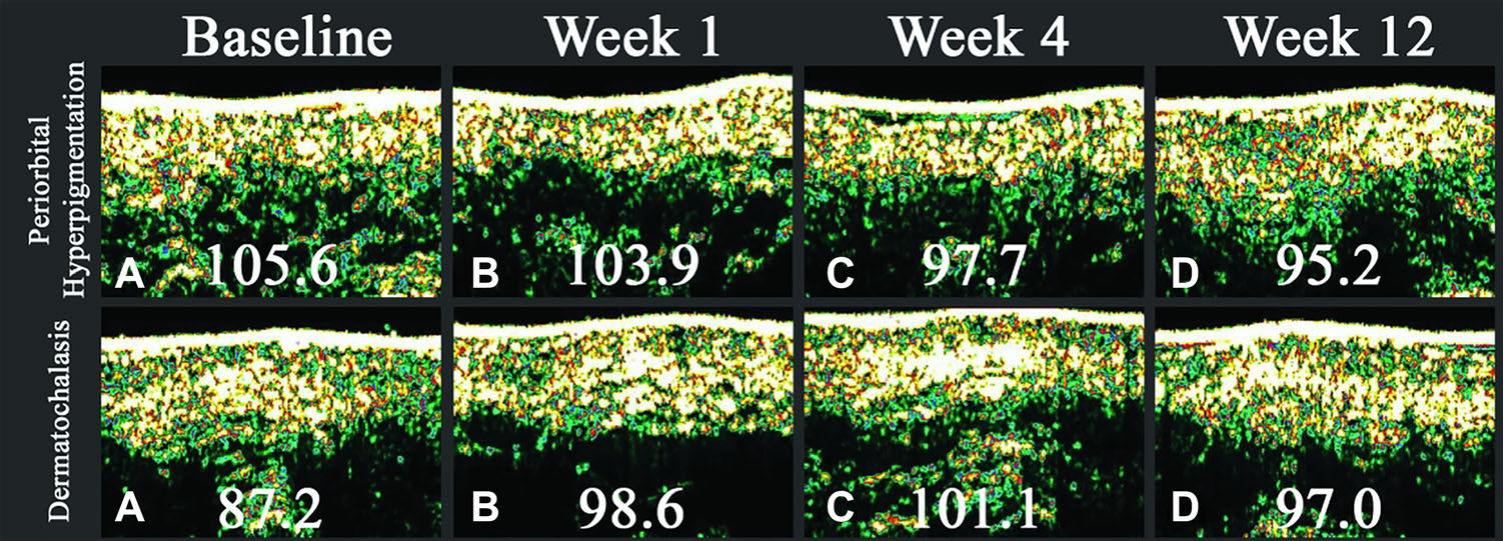
Image produced from the ultrasound probe of DermaLab® Combo for the same periorbital hyperpigmentation and dermatochalasis patients in Fig. 2 and 2, **A** before receiving treatment, **B** 1 week after injection, **C** 4 weeks after injection, and **D** 12 weeks after injection. Intensity scores (of arbitrary units, indicating ultrasound signal intensity) are shown for each study. Though the calculated intensity score had decreased throughout the 12 weeks for the periorbital hyperpigmentation patient, signal intensity seems relatively stable, and the thickness of the dermis did not change substantially. On the other hand, signal intensity had increased noticeably for the dermatochalasis patient, with mild tapering of effects at week 12. Increased thickness of the dermis is also observed

The dermatochalasis group (Table 1) showed a significant increase in skin hydration following the injection, with a mean difference of 46.98 μS observed at week 1 (*p* = 0.012). Although the levels remained elevated at week 12, the difference became statistically insignificant. Skin density exhibited an initial increase (*p* = 0.171), reaching its peak at week 4 (*p* = 0.014), followed by a gradual decrease by week 12, yet still maintaining higher levels compared to the baseline (*p* = 0.110). The alterations in skin density are easily discernible in the ultrasound images (Fig. 4). The color lightness in the CIELAB color space exhibited a notable rise in color lightness (*p* = 0.033), which gradually decreased by week 4 (*p* = 0.238). The viscoelasticity of the skin exhibited a reduction of 14.29% by week 4 (*p* = 0.001) and sustained a decrease of 11.64% by week 12 (*p* = 0.014).

**Table 1:** Skin hydration, elasticity, lightness index, skin density, and skin thickness of test subjects diagnosed with dermatochalasis, measured at 1 week, 4 weeks, and 12 weeks post-procedurally. The number represented both eyes from six participants. The notation n=12 indicates that data were collected from both eyes of six participants in each group

Dermatochalasis (n = 12**)**	Hydration (μS)	L* (CIELAB)	Density (intensity score)	Viscoelasticity (%)
Baseline	290.33 ± 49.65	32.3 ± 2.6	90.9 ± 13.0	66.03 ± 13.70
Week 1	337.31 ± 94.24*	33.5 ± 2.43*	94.2 ± 17.6	66.34 ± 12.50
Week 4	325.73 ± 67.82*	32.8 ± 1.8	97.6 ± 17.3	51.74 ± 7.02*
Week 12	323.67 ± 57.33	32.6 ± 2.5	96.3 ± 14.3	54.39 ± 7.47*

*Indicates a statistically significant difference from baseline measurements (*p* < 0.05).

The periorbital hyperpigmentation group (Table 2) exhibited an initial decrease in skin hydration of 3.96 μS at week 1 (*p* = 0.112), followed by a gradual increase over time. By the 12th week, skin hydration was significantly increased by 82.90 μS (*p* = 0.001). Skin density exhibited a consistent rise over the 12-week period; however, these alterations were deemed statistically insignificant. The CIELAB lightness level exhibited a notable increase at week 1 (*p* = 0.016) and week 4 (*p* = 0.042), followed by a decline at week 12 (*p* = 0.094). Skin viscoelasticity exhibited an 11.78% decrease at week 4 (*p* = 0.002), stabilizing by week 12 with a 9.96% reduction (*p* = 0.002).

**Table 2:** Skin hydration, elasticity, lightness index, skin density, and skin thickness of test subjects diagnosed with peri-orbital hyperpigmentation, measured at 1 week, 4 weeks, and 12 weeks post-procedurally. The notation n=12 indicates that data were collected from both eyes of six participants in each group

Periorbital hyperpigmentation (n = 12)	Hydration (μS)	L* (CIELAB)	Density (Intensity Score)	Viscoelasticity (%)
Baseline	264.73 ± 37.11	31.1 ± 4.5	97.2 ± 20.6	65.12 ± 10.12
Week 1	260.77 ± 58.42	33.4 ± 2.8*	99.2 ± 13.0	62.07 ± 12.08
Week 4	277.60 ± 59.97	32.6 ± 2.8*	99.9 ± 16.1	53.34 ± 7.55*
Week 12	347.62 ± 66.35*	32.6 ± 2.6	100.9 ± 18.4	55.16 ± 6.16*

*Indicates a statistically significant difference from baseline measurements (*p* < 0.05)

**Fig. 5:**
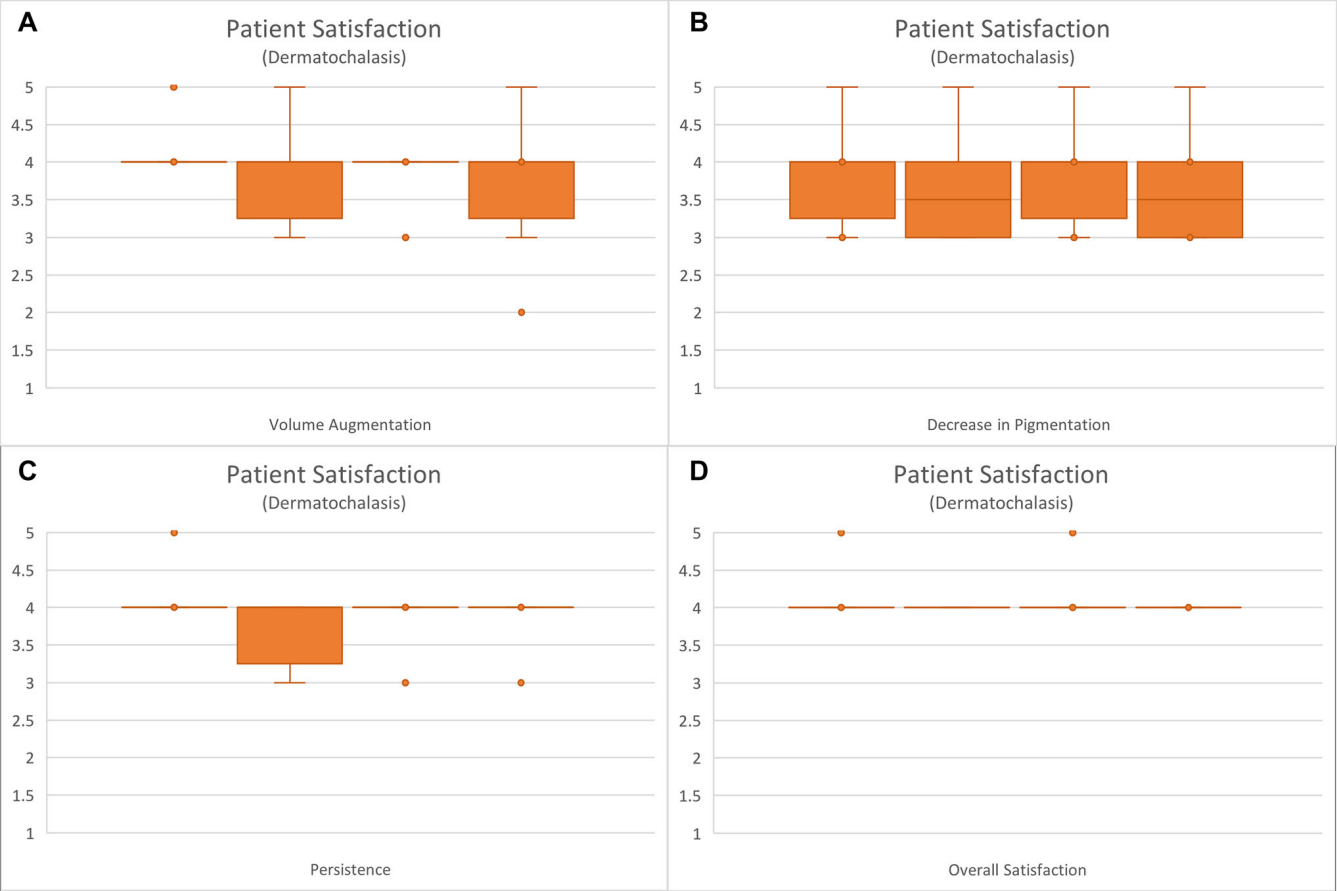
Patient satisfaction in the dermatochalasis group for **A** volume augmentation, **B** decrease in pigmentation, **C** persistence, and **D** overall satisfaction

**Fig. 6:**
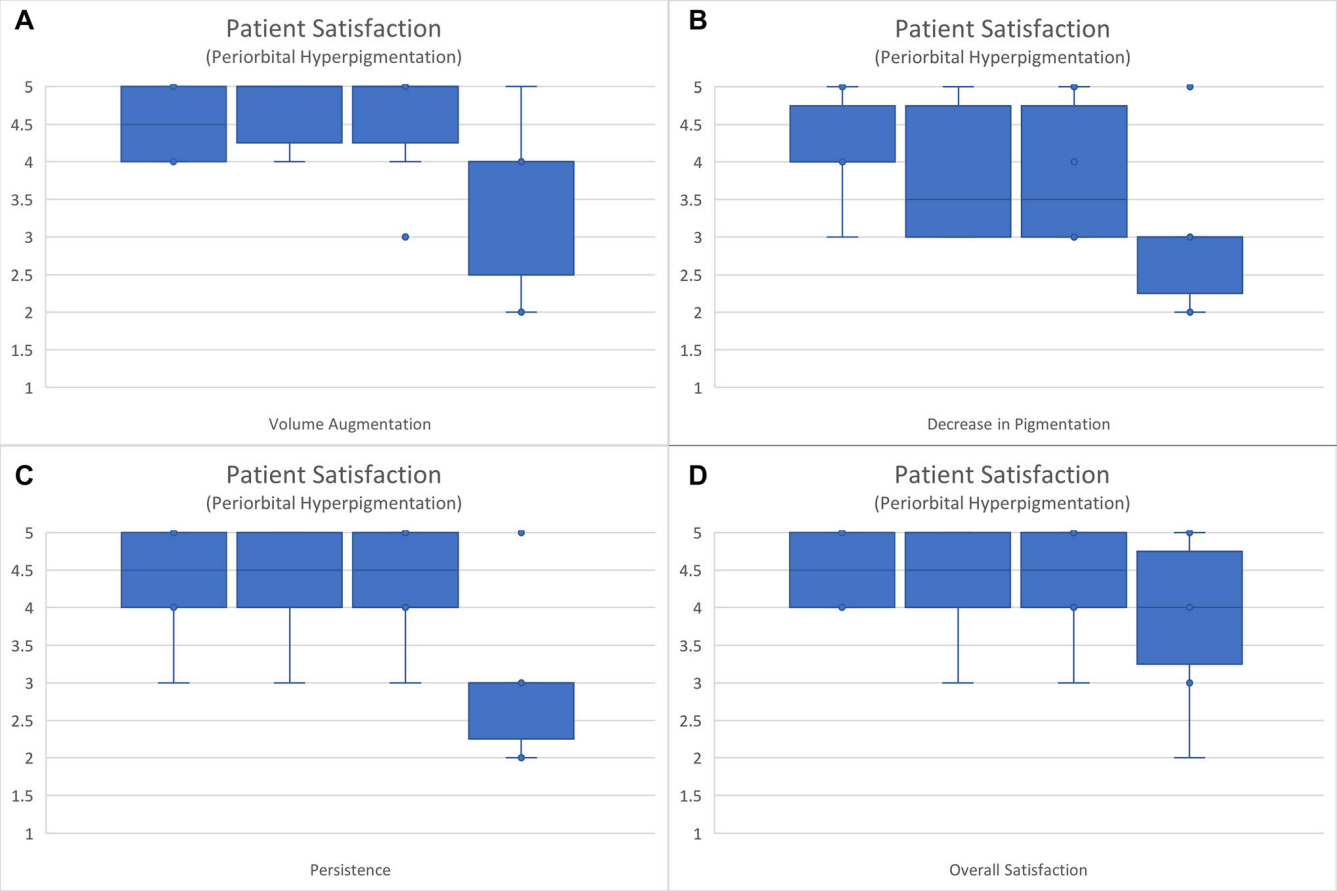
Patient satisfaction in the periorbital hyperpigmentation group for **A** volume augmentation, **B** decrease in pigmentation, **C** persistence, and **D** overall satisfaction

Patient satisfaction was generally positive for both groups initially. At both the immediate post-injection assessment and at week 1, the median overall satisfaction score for the dermatochalasis group was 4, while for the periorbital hyperpigmentation group, it was 4.5. By the 12th week, a reduction in volume augmentation, pigmentation, persistence, and overall satisfaction was observed. The Kruskal-Wallis statistics performed for both groups (Figs. 5 and 6) did not reveal any statistical significance in the comparison of patient satisfaction levels between those recorded during follow-up and those reported immediately post-procedure (Table 3).

**Table 3:** Statistical analysis via the Kruskal-Wallis test for patient satisfaction results obtained immediately post-procedurally and at 1 week, 4 weeks, and 12 weeks post-procedurally. The notation n=6 refers to the satisfaction ratings provided by the six participants in each group

	Kruskal-wallis test (*p*-value)	
	Dermatochalasis (n = 6)	Periorbital hyperpigmentation (n = 6)
Volume augmentation	0.7807	0.3156
Decrease in pigmentation	0.9536	0.2498
Persistence	0.5938	0.1239
Overall satisfaction	0.9484	0.7819

Regarding post-injection complications, one participant experienced bruising that had not yet resolved by the first day following the injection. During the same follow-up period, three participants reported pain; one experienced redness and itching, while another reported a sensation of heat at the injection site.

By the end of the first week, most immediate reactions had subsided. However, one participant reported experiencing a sensation akin to a foreign body, while another described a lump-like feeling. This lump-like sensation emerged in one participant during the fourth week. By the twelfth week, no participants reported any allergic reactions or complications. Overall, the complications were generally mild and transient, with most resolving within the first week following the injection.

## Discussion

Currently, the mainstay of treatment for laxity and redundancy of the periorbital tissue and overlying skin includes surgical, volume augmentation, and skin rejuvenation [15–17]. For less severe cases or patients who desire a less-invasive approach, dermal fillers can be used to help fill the volume defect caused by aging. Treatments for periorbital hyperpigmentation on the other hand include lasers that target pigment and vascularity, autologous fat transplant, platelet-rich plasma injection, dermal fillers, or topical agents such as phenolic or nonphenolic bleaching agents, hydroquinone, or kojic acid [1, 18]. Each treatment methodology is associated with its own advantages and shortcomings.

Over the last 10 years, there has been an increase in the use of cross-linked collagen sourced from pigs, demonstrating comparable results to traditional bovine fillers. Furthermore, it demonstrates decreased immunogenicity and increased resilience to collagenolytic degradation [19, 20]. The feasibility and efficacy of these cross-linked porcine fillers have been demonstrated in both animal models and human subjects [12, 19, 21, 22]. In our study, we employed a glutaraldehyde cross-linked, highly purified, specific pathogen-free porcine dermal type I atelocollagen infused with lidocaine as the preferred dermal filler for our research. Cross-linked collagen has demonstrated efficacy in various applications, such as addressing fecal incontinence and sphincter insufficiency, urinary incontinence, orbital augmentation in anophthalmic patients, management of vocal fold immobility, and cosmetic procedures for the face [23–29].

Presently, research focusing on periorbital rejuvenation and dermatochalasis has been constrained to various treatment approaches, including surgical intervention, dermal fillers, autologous fat grafting, botulinum toxin administration, platelet-rich plasma therapy, and laser treatments. In the context of periorbital rejuvenation, the tear trough area is frequently targeted for enhancement. The injection of hyaluronic acid dermal fillers is a popular choice for correcting tear trough deformities [10, 31, 32]. The administration methods for hyaluronic acid in this area are primarily based on individual preference; however, there is a consensus that the filler is typically injected either in the pre-periosteal plane or within the orbicularis oculi muscle itself. The rationale for choosing these specific areas for injection is based on the potential consequences of injecting hyaluronic acid too superficially. Superficial injection may result in visible lumps in the overlying tissue and a noticeable blue discoloration [31, 32]. Despite preventive measures, the occurrence of lumps persists in numerous patients, necessitating correction through either manual manipulation or hyaluronidase injection [31] .The study showcased the effectiveness of collagen dermal fillers as a viable alternative for managing dermatochalasis and periorbital hyperpigmentation in patients. Collagen dermal fillers exhibit lower susceptibility to lumping, enabling their more superficial injection for precise adjustment of skin contour. Given the fewer precautions associated with collagen-based fillers, the learning curve is notably less steep in comparison to hyaluronic acid fillers.

The retaining ligaments of the face are fibrous tissues that serve as anchors, tethering the overlying dermis and soft tissue to the underlying denser connective tissue and bone. This anchoring function is essential for maintaining their normal anatomical position [33–35]. In the study by Chuang et al., a technique known as True Lift™ involves the identification and injection of hyaluronic acid at the base of the osteocutaneous facial retaining ligaments, which tightens them through a cantilever effect. Theoretically, the hyaluronic acid serves as a support for the retaining ligaments, resulting in the indirect lifting of the overlying soft tissue and improving skin sagging and laxity [36]. Our deep collagen injection aims to offer improved support to the tear trough ligaments, rather than merely volume augmentation.

Pitaru et al. (2007) discovered that glutaraldehyde-cross-linked collagen dermal fillers, in comparison to non-cross-linked collagen dermal fillers, retained some implant materials 12 months post-injection [20]. Nonetheless, both categories of dermal fillers experienced a loss of their initial shape over time, with glutaraldehyde-cross-linked fillers showing a loss of the original shape 6 months after injection. The desired volumizing effects of glutaraldehyde-cross-linked collagen are achieved through direct augmentation of the dermis. It is anticipated that the degradation of the original shape would lead to the loss of these effects [26]. Our study demonstrates a similar pattern, with the impacts on viscoelasticity and skin density reaching their maximum at week 4 and diminishing by week 12. A touch-up injection between weeks 4 and weeks 12 is recommended to sustain the desired effects by the end of this period.

Given that tyrosinase plays a crucial role in melanogenesis, evaluating tyrosinase activity can serve as a method to measure the anti-melanogenic impact of different substances [37]. Collagen hydrolysates, which are the degradation products of collagen, have been suggested to function as tyrosinase inhibitors. Hong et al. (2019) discovered that porcine collagen hydrolysates exhibit restricted tyrosinase inhibitory effects. Conversely, research on collagen sourced from other organisms has demonstrated encouraging melanogenesis-inhibitory properties [38–41]. Based on the quantitative data obtained in our study, the skin color exhibited an elevation in lightness among patients with dermatochalasis and periorbital hyperpigmentation following the administration of porcine collagen. This suggests a potential reduction in melanogenic activity. Studies on porcine collagen hydrolysates are currently limited, necessitating further investigation to establish a more definitive relationship between collagen and its by-products and their potential inhibition of melanogenesis.

Following the administration of a dermal filler, the patient’s evaluation typically involves a visual assessment, with the results being qualitatively documented. In the context of evaluating dermatochalasis and periorbital hyperpigmentation, skin hydration, density, elasticity, and color are crucial factors of interest. In current literature, DermaLab® Combo and Cutometer® MPA 580 have primarily been utilized for evaluating scar and burn wounds. However, their application for comparing pre-procedural and post-procedural outcomes in periorbital cosmetic surgery has not been extensively addressed or embraced [42–45]. The utilization of a multi-parameter skin analysis system offers a more objective method for evaluating the impacts of comparable cosmetic procedures. In addition to statistical analyses, the feasibility of inter-study comparisons is enhanced by the absolute nature of the measurements. The utilization of skin analysis systems enhances the reliability of evaluating the effectiveness of cosmetic procedures and facilitates the generation of valuable data for future use.

A high dropout rate of 40% was observed in each group. This study has adopted a patient-centric perspective by incorporating satisfaction data to address this issue. Despite the challenges encountered, particularly the small sample size and elevated dropout rate, our research focuses on Asian individuals, thereby providing a valuable reference for clinical applications.

The marginal enhancements documented in this study could be elucidated by the restricted sample size resulting from the loss of patient follow-up and the relatively youthful age of the participants. In young patients, the severity of dermatochalasis and periorbital hyperpigmentation varies from mild to moderate, leading to restricted enhancements. Recruiting patients of advanced age or with more severe disease could enhance the robustness of the study findings. Additionally, our participants with dermatochalasis were younger; however, severe cases of dermatochalasis still necessitate surgical revision. Therefore, relying solely on injections to improve the condition may not significantly motivate participants to join the study. Furthermore, the extended time interval between measurements at weeks 4 and 12 has resulted in significant reductions in certain metrics. An additional assessment at the 8-week mark could help establish a more accurate schedule for touch-up injections. Following patients for more than 12 weeks is also recommended to ensure the sustainability of the effects.

## Conclusions

The utilization of skin analysis systems for evaluating patients undergoing dermal filler injections offers physicians quantitative data that can serve as an indicator for determining the necessity of subsequent touch-up procedures. Based on the quantitative data obtained in this study, the injection of cross-linked porcine collagen dermal filler can serve as a minimally invasive yet highly effective procedure for enhancing skin laxity in dermatochalasis and addressing discoloration in periorbital hyperpigmentation. Patients exhibit high satisfaction levels with this treatment; however, periodic touch-up sessions may be necessary to sustain its efficacy.
